# Incidence of neutropenia, chemotherapy delivery, and use of colony-stimulating factor in patients with non-Hodgkin lymphoma of different age groups

**DOI:** 10.3109/10428194.2011.555023

**Published:** 2011-02-11

**Authors:** Ruth Pettengell, Matthias Schwenkglenks

**Affiliations:** 1 Cellular and Molecular Medicine, St. George's University of London, London, UK; 2 Institute of Pharmaceutical Medicine, University of Basel, Basel, Switzerland

Chemotherapy regimens used to treat non-Hodgkin lymphoma (NHL), such as cyclophosphamide, dox-orubicin, vincristine, and prednisone (CHOP), with or without rituximab, are associated with a high (≥20%) risk of febrile neutropenia (FN) [1]. Older age is an additional risk factor for FN [1,2], and existing comorbidities increase FN-associated mortality [3].

Neutropenic complications are potentially life-threatening [4] and represent the most frequent dose-limiting toxicity of myelosuppressive chemotherapy [1,5], often resulting in treatment delays and dose reductions [6]. For low relative dose intensity (RDI), which is especially common among older patients with NHL [7], an association with poorer outcomes has been reported [8], emphasizing that when a curative regimen has been selected and planned, there should be as little deviation from the planned dose intensity as possible. Colony-stimulating factors (CSFs) have been shown to reduce the incidence and severity of neutropenic events across a broad range of malignancies and regimens, supporting the delivery of full chemotherapy dose intensity [1,5,9].

The Impact of Neutropenia in Chemotherapy -European Study Group (INC-EU) prospective, observational study previously reported the incidence and risk of chemotherapy-induced neutropenia (CIN), FN, and dose modifications in patients with breast cancer and lymphoma [10]. In comparison to patients with breast cancer, those with lymphoma experienced higher incidences of FN and grade IV neutropenia. Here we present results from a sub-analysis of patients with NHL, assessing the impact of age on the frequency of neutropenic events, chemotherapy delivery, and CSF use.

The INC-EU prospective, observational study enrolled 749 patients with breast cancer or lymphoma initiating a new course of chemotherapy during 2004-2005, recruited from 66 clinical centers in Belgium, France, Germany, Spain, and the UK [10].

Inclusion and exclusion criteria for this study have been previously reported in detail [10]. For this subgroup analysis, patients with NHL (*n* = 240) were divided into three age groups: ≤60 years (*n* = 84), 61-70 years (*n* = 77), and > 70 years (*n* = 79). The primary outcome measure was incidence of CIN; secondary outcome measures were incidence of FN, patterns of chemotherapy delivery (dose delays/ reductions, RDI, and non-completion), and use of CSFs and risk of death (in the presence and absence of FN). Grade IV neutropenia was defined as an absolute neutrophil count (ANC) <0.5 × 10^9^/L and FN as grade IV neutropenia and temperature >38°C. CSF use was defined as primary prophylaxis (CSF use in the first cycle before a documented grade III-IV CIN occurred or denoted as primary prophylaxis by study site) or reactive use (secondary CSF prophylaxis in cycles other than the first, or CSF used as treatment). Baseline, treatment, and outcome parameters were summarized by age group and compared. The temporal relationship of dose delays with episodes of grade IV CIN and FN was assessed. Causes of death and treatment discontinuation in the highest age group were evaluated on a patient-by-patient basis.

Baseline characteristics were comparable between groups, except for parameters known to vary with age (Table I). The incidence of cardiovascular and cardiac comorbidities, lactate dehydrogenase > 500 IU/L, and glucose > 160 mg/dL (8.8 mmol/L) increased with age, and the glomerular filtration rate decreased with age. In addition, the proportion of female patients increased with age.

**Table I:** Patient and disease characteristics.

Characteristic	≤60 years (*n* = 84)	61–70 years (*n* = 77)	> 70 years (*n* = 79)
Age in years: mean ± SD	49.0±9.5	65.9±3.1	75.9±4.4
Female gender: pts (%)	28 (33.3)	33 (42.9)	44 (55.7)
Body surface area*: mean±SD	1.89±0.19	1.84±0.16	1.78±0.18
IPI index: pts† (%) Low (0–1)	42 (51.2)	16 (20.8)	17 (21.8)
Intermediate (2–3)	35 (42.7)	49 (63.6)	48 (61.5)
High (≥4)	5 (6.1)	12 (15.6)	13 (16.7)
Glomerular filtration rate in mL/min‡: mean±SD	105±31	79±25	63±18
ANC <1.5 × 10^9^/L: pts† (%)	4 (4.9)	4 (5.4)	2 (2.6)
Cardiovascular comorbidity: pts (%)	14 (16.7)	18 (23.4)	33 (41.8)
Cardiac comorbidity: pts (%)	7 (8.3)	10 (13.0)	15 (19.0)
Liver comorbidity: pts (%)	1 (1.2)	2 (2.6)	2 (2.5)
Renal comorbidity: pts (%)	3 (3.6)	7 (9.1)	6 (7.6)

* Calculated using the Mosteller formula.

† *n* < expected due to missing values.

‡ Estimated using the Cockroft–Gault formula.

SD, standard deviation; pts, patients; ANC, absolute neutrophil count.

The majority of patients received CHOP, either on a 3-weekly (74%) or 2-weekly (17%) schedule. The proportion of patients with 2-weekly CHOP increased with age (8%, 17%, and 27% of patients aged ≤60, 61-70, and > 70 years, respectively). For most patients, six cycles of chemotherapy were planned across all age groups. In the ≤60, 61-70, and > 70 year-old groups, 83%, 83%, and 79% of patients, respectively, received rituximab.

**Figure 1:**
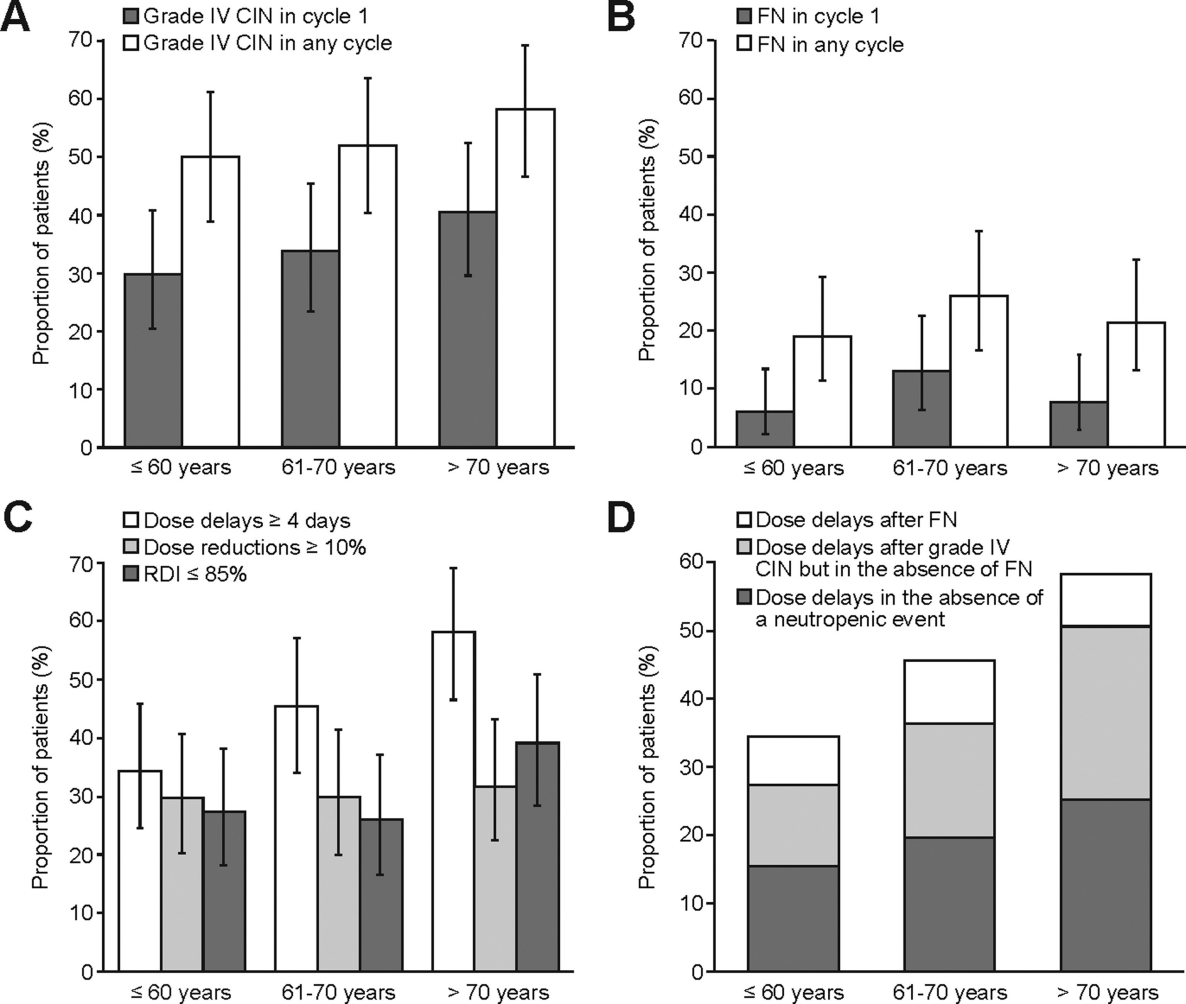
Incidence of CIN and dose modifications with age. Incidence of (A) grade IV CIN (ANC < 500/mm^3^ or, if ANC unavailable, white blood cell count < 1000/mm^3^) and (B) FN (grade IV CIN plus fever [≥38°C]) in cycle 1 and in any cycle. Error bars represent 95% CIs. (C) Proportion of patients experiencing dose delays ≥4 days, dose reductions ≥ 10%, and RDI ≤ 85%. Non-administered cycles were taken into account to calculate dose reductions and RDI, except in the case of patient death. Error bars represent 95% CIs. (D) Proportion of patients experiencing dose delays ≥4 days after FN, after grade IV CIN but in the absence of FN, and in the absence of a neutropenic event. CIN, chemotherapy-induced neutropenia; ANC, absolute neutrophil count; FN, febrile neutropenia; CI, confidence interval; RDI, relative dose intensity.

Grade IV CIN and FN occurred frequently in all age groups. Overall, 54% of patients experienced grade IV CIN; there was a trend toward an increased incidence with age [Figure 1(A)]. Twenty-two percent of patients developed FN in any cycle; the incidence did not increase with age [Figure 1(B)].

The relative risk (RR) of death for patients who experienced FN in any cycle, versus those without FN, was 1.7 for the younger age groups combined (95% confidence interval [CI] 0.2-18.6; *p* = 0.535). For patients > 70 years, the RR was 10.9 (95% CI 2.4-49.4; *p* = 0.001), suggesting that although the incidence of FN did not increase with age, patients in this age group were at increased risk of death from FN.

Overall, 28% of patients received primary CSF prophylaxis and 29% of patients required other CSF use. Use of daily CSF (filgrastim) was highest in patients aged 61-70 years; pegfilgrastim was used most frequently in patients > 70 years. The proportion of patients receiving primary CSF prophylaxis was comparable across all age groups (24%, 29%, and 30% of patients aged ≤ 60, 61-70, and > 70 years, respectively). Reactive CSF use was seen frequently and to a similar extent in each age group (32%, 25%, and 29% of patients aged ≤ 60, 61-70, and >70 years, respectively).

Patients in the oldest age group were less likely to complete their planned treatment: 27% of patients >70 years did not complete their planned chemotherapy, as compared to 19% and 14% of patients aged ≤60 and 61-70 years, respectively. Of the non-completers >70 years, 48% discontinued due to adverse events, 38% died, and 14% withdrew consent or were discontinued due to non-compliance. In this age group, 90% of adverse events leading to discontinuation and 88% of deaths involved CIN, FN, or infection.

The occurrence of dose delays, dose reductions, and RDI ≤ 85% is shown in Figure 1(C). The proportion of patients with dose delays ≥ 4 days increased with age. Figure 1(D) shows the proportion of dose delays that occurred in the absence of neutropenic events, after grade IV CIN, or after FN. With increasing age, a greater proportion of patients experienced dose delays in the absence of neutrope-nia, and in the presence of grade IV CIN without FN. The proportion of patients with dose reductions ≥10% was comparable across age groups. RDI ≤85% occurred in 30% of patients overall, and was most frequent in patients > 70 years (almost 40% of this group) [Figure 1(C)].

The high incidence of RDI ≤85% in patients >70 years appears to have been driven by an increased number of dose delays in these patients, which may have protected against FN, possibly explaining why FN rates in this group were not higher than the overall average. This potential protective effect of dose delays was not attributable to pre-planned decreases in RDI, which would have been recorded separately, as per study protocol.

Although rates of FN were similar across age groups, myelosuppression impacted most strongly on outcomes in patients > 70 years, who were at higher risk of FN-related mortality and more likely to discontinue treatment due to CIN, FN, and infectious complications. Although most elderly patients with cancer can tolerate standard chemotherapy regimens [5], our data suggest that patients >70 years are at higher risk of a worse outcome following a neutropenic event. Appropriate supportive care and close clinical monitoring are therefore of particular importance for this population. Results of a recent integrated analysis suggested that elderly patients with breast cancer (≥65 years) benefited from primary prophylaxis with pegfilgrastim: the incidence of FN, dose reductions, and FN-related hospitaliza-tions was reduced in these patients, in comparison to those receiving current practice neutropenia management [11].

Despite the fact that the European Organisation for Research and Treatment of Cancer (EORTC) guidelines for the elderly recommend that prophylactic CSF is given to all patients > 65 years receiving myelotoxic chemotherapy [12], there was no increased use of CSF in patients >70 years as compared to younger patients, either as primary prophylaxis or reactive use. A considerable proportion of patients required CSF as secondary prophylaxis or treatment. This, together with the overall high FN rate and low RDI, may suggest a false economy: where primary CSF prophylaxis is not given, a substantial proportion of patients require reactive initiation of CSF during their treatment course.

In summary, CIN and FN were considerable in patients with NHL across all age groups. Although FN rates were similar across all age groups, myelosuppression had the greatest impact on patients >70 years, suggesting that CSF prophylaxis may be particularly relevant for this population.
